# Neonatologist performed lung ultrasound: NPLUS—proposal for a consistent ultrasound terminology

**DOI:** 10.3389/fped.2022.1007672

**Published:** 2023-02-14

**Authors:** Lukas Aichhorn, Erik Küng, Bernhard Schwaberger

**Affiliations:** ^1^ Division of Neonatology, Paediatric Intensive Care & Neuropaediatrics, Department of Paediatrics and Adolescent Medicine, Comprehensive Center for Paediatrics, Medical University of Vienna, Vienna, Austria; ^2^ Division of Neonatology, Department of Paediatrics and Adolescent Medicine, Medical University of Graz, Graz, Austria

**Keywords:** lung ultrasound, POCUS, neonatology, neonatologist performed lung ultrasound, NPE

## Introduction

The increasing number of publications regarding neonatal lung ultrasound and its use will have a positive impact on the clinical care for our small patients, since lung ultrasound—as a point-of-care ultrasound (POCUS) exam—has proved its worth as a safe, quick, and reliable diagnostic tool (1). However, there is an ongoing discussion on who should perform ultrasound exams in which situations. We read with great interest the recent review article by Nestaas, who accomplished to thoroughly summarize the main aspects of neonatologist performed echocardiography (NPE) in the newborn (2), a term that was firstly introduced in 2016 by the European Society for Paediatric Research (ESPR) and the European Society for Neonatology (ESN) (3). On the other hand, we followed the discussion that evolved around Andronikou et al. and van Rijn et al. after expressing their concerns regarding *non-radiologist POCUS*, a term that we will also use in this statement (4–7).

### Neonatal lung ultrasound

In recent years, more and more literature focused on the application of ultrasound for specific patient groups, interventions, or clinical problems. In the pediatric field, neonatal lung ultrasound has been one of the most thoroughly studied applications in the last several years and as a result, it is now included in a recently published POCUS guideline issued by the European Society of Paediatric and Neonatal Intensive Care (ESPNIC) (8).

Neonatal lung ultrasound has not only been proven to be safe and relatively easy to learn, but by now enables us to guide and refine our therapies. In particular, evidence shows that many of the main reasons for respiratory distress in the newborn, including pneumothorax, pleural effusion, respiratory distress syndrome, transient tachypnoea of the newborn, pneumonia and atelectasis can be diagnosed by lung ultrasound (1, 9, 10). Another prominent application is the use of a lung ultrasound score, a semiquantitative method to assess the neonatal lung (11). Based on this score, strong evidence allows us to identify infants who need surfactant, or even a second dose of surfactant (11, 12), infants who are very likely to develop bronchopulmonary dysplasia (13), or infants who suffer from lung edema after cardiac surgery (14). In our opinion, these insights are not only highly relevant for the clinician, but they also have an immediate clinical consequence and are therefore primarily performed by the attending neonatologist. The direct positive impact of these applications reflects what makes POCUS so powerful in clinical practice.

### NPLUS—neonatologist performed lung ultrasound

Based on these developments and discussions, we would like to submit a proposal concerning the terminology of the lung ultrasound examination in newborn patients. In accordance with the well-established term NPE, we changed the terminology in our units to “Neonatologist Performed Lung Ultrasound” (NPLUS). This abbreviation, which was first introduced in one of our most recent publications, allows for a proper classification of the investigation (10). The term NPLUS emphasizes the functional and integrative character of the examination as a POCUS technique, as it is performed at the bedside by the involved neonatologist, who interprets the results in consideration of the patient's clinical condition.

Some authors, including Lichtenstein, who invented the concept of lung ultrasound and is recognized as the “father” of lung ultrasound, argued that the abbreviation LUS should only be used for “lung ultrasound *score*”, and the abbreviation LU should be used for “lung ultrasound” (12, 15, 16). However, use of LU and LUS appears to be inconsistent among dozens of publications, which again demonstrates the need for a discussion regarding the nomenclature by special interest groups and scientific societies.

**Figure 1:**
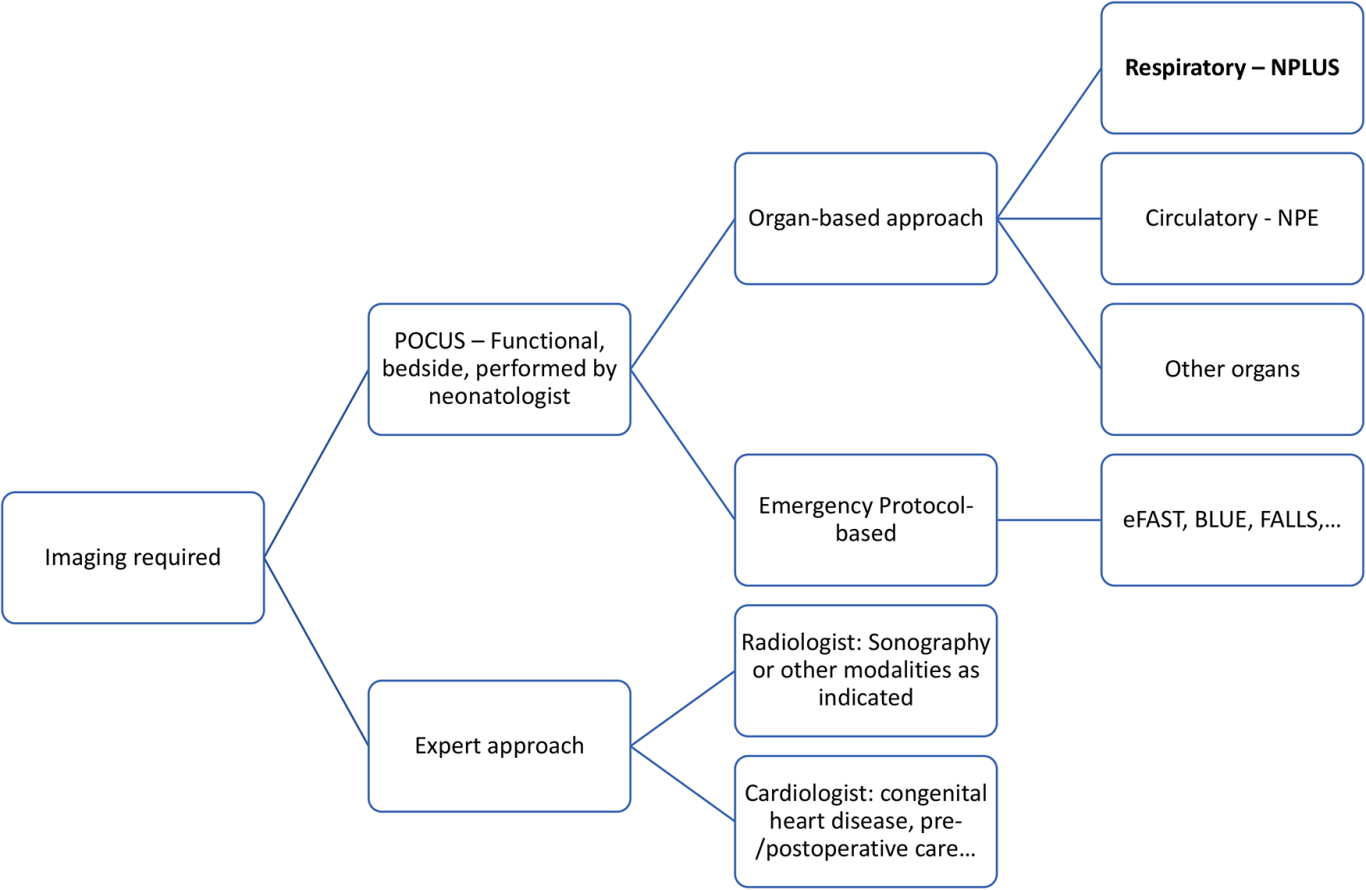
Decision tree for sonographic imaging of neonates. Two approaches (POCUS and expert approach) are further investigated. At the bedside, POCUS may provide quick answers regarding specific, organ-related questions by scanning the corresponding region or by application of an emergency protocol (e.g., respiratory insufficiency, intracranial haemorrhage, cardiac tamponade). In analogy to NPLUS and NPE, other organ-based exams might follow this nomenclature. Expert approach allows for a more thorough and sophisticated diagnostic evaluation, but might be more time-consuming and depends on the availability of skills and resources.

In our opinion, the term NPLUS (or alternatively NPLU) helps differentiate a lung POCUS examination from a possibly more standardized and thorough assessment of the lung performed by a radiologist. This terminology may be extended to further applications of neonatologist performed ultrasound (abdominal, cranial, spinal ultrasound) allowing for a harmonization of ultrasound terminology and communication in neonatology (see Figure 1). The introduction of consistent terminology facilitates the difficult but inevitable process of standardization.

## Discussion

The introduced distinction might calm down the discussion about who should perform lung ultrasound and who should not. It is of particular importance for the clinician in pediatrics to appreciate the value of expert radiologists and acknowledge the limitations of non-radiologist ultrasonography in order to avoid errors whenever possible, and subsequently provide the best care for our patients. We could not agree more with the main points of Arthurs et al., who emphasize the importance of experience, training, and establishment of a learning environment to increase the quality of ultrasound examinations (7).

The increasing use of lung ultrasound was initiated primarily by intensivists, leading to a majority of publications in pediatric journals. As Tomà states in his 2020 review, “fewer than 10% of the articles on lung ultrasonography, in the last 10 years, have been published in radiologic journals” (17). Of course, the possible differences in peer-review processes between radiologic and pediatric journals are obvious and must not be ignored. However, both radiologists and pediatricians acknowledge the need for standardized training and guidelines (18–20).

Concerns regarding non-radiologist POCUS were raised as soon as medical specialties recognized the potential of ultrasound (US) for different applications, most prominently seen in adult emergency care, but later also in pediatrics and other fields, possibly “undermining the role of the radiologist” (4, 21). However, we believe that both neonatologist- and radiologist performed ultrasound contribute to the care of our small patients, while each approach has various strengths and limitations in different situations, as the previous authors who are mentioned above correctly stated.

Furthermore, in our experience, POCUS training is often provided without radiologists being involved. Hence, special attention on understanding the limitations of non-radiologist POCUS or—maybe even more important –training programs held jointly involving radiologists and various clinical specialists seem crucial.

We do recognize that a standardization of the term should be discussed and implemented by a joint discussion led by scientific societies. Two recent publications in this field can be considered as examples for such joint efforts, namely the “International evidence-based recommendations for point-of-care lung ultrasound” by the International Liaison Committee on Lung Ultrasound (ILC-LUS) and the “International evidence-based guidelines on Point of Care Ultrasound (POCUS) for critically ill neonates and children” issued by the POCUS Working Group of the European Society of Paediatric and Neonatal Intensive Care (ESPNIC) (8, 9). In our opinion, the latter organization can serve as a suitable forum to come to a decision in this matter. From a radiological standpoint, however, the European Federation of Societies for Ultrasound in Medicine and Biology (EFSUMB), which regularly publishes guidelines and recommendations, also offers a platform for joint sessions and discussions regarding innovations in sonography, hence there might be a significant participation by the organization in the discussion evolving around this proposal (22). Lastly, the P2Network published consensus papers regarding application and training in Pediatric Emergency Medicine Ultrasound and is a platform of clinical experts in POCUS (23).

We would endorse a harmonization of the terminology by establishing “NPLUS” in research papers in this field.
